# Ossiculoplasties: à propos de 30 cas et revue de la littérature

**DOI:** 10.11604/pamj.2021.38.187.27449

**Published:** 2021-02-18

**Authors:** Khalid Bouhafs, Azeddine Lachkar, Abderrahim Benallal, Drissia Benfadil, Mohammed Rachid Ghailan

**Affiliations:** 1 Service d'Oto-Rhino-Laryngologie et de Chirurgie Cervico-Faciale, Centre Hospitalier Universitaire Mohammed VI d'Oujda, Oujda, Maroc

**Keywords:** Lyse ossiculaire, ossiculoplasties, résultats fonctionnels, Ossicular lysis, ossiculoplasties, functional results

## Abstract

L'objectif était d'évaluer les résultats fonctionnels des ossiculoplasties. Il s'agissait d'une étude rétrospective, au sein du service d'otorhinolaryngologie (ORL) et de chirurgie cervico-faciale (CCF) au centre hospitalier universitaire (CHU) Mohammed VI d'Oujda, étalée sur une durée allant du mois d'octobre 2018 au mois de mars 2020, ayant pu regrouper 30 patients parmi 45 otites séquellaires qui ont été opérés pour ossiculoplastie par une voie transméatale endoscopique. La moyenne d'âge des patients était de 31,8 ans avec un sex-ratio F/H de 1,5. L'exploration de la caisse du tympan a montré, selon la classification d'Austin, 18 cas type A, 5 cas type B, 3 cas type C et 4 cas type D. Soixante-dix pourcent (70%) des oreilles opérées avaient un déficit audiométrique entre 30 et 40 dB et 30% des patients ont un déficit audiométrique ≥40 dB. L'ossiculoplastie type II a été la plus pratiquée dans 26 cas avec utilisation du cartilage tragal dans 18 cas, interposition d'enclume autologue et mise en place d'une PORP en titane dans 4 cas pour chacune alors que l'ossiculoplastie type III a été pratiquée dans 4 cas avec mise en place d'une opération de reconstruction totale des osselets (TORP) en titane. Le groupe d'ossiculoplastie type II avait un gain auditif ≥20 dB dans tous les cas alors que celui d'ossiculoplastie type III ne l'avait que dans un seul cas. Le taux de réussite était de 90%. L'analyse de ces résultats montre que globalement les résultats sont légèrement moins bons dans les ossiculoplasties type III que dans les ossiculoplasties type II. Cependant, les études comparatives entre différents montages, entre prothèses et autogreffes ou entre différents types de matériaux n'objectivent que des différences minimes pour un même auteur ou pour une même équipe. Il semble pour beaucoup d'auteurs que la préservation du manche du marteau permette de meilleurs résultats fonctionnels.

## Abstract (en)

The purpose of this study was to evaluate the functional outcomes of ossiculoplasties. We conducted a retrospective study, in the department of ear, nose and throat (ENT) and congestive cardiac failure (CCF) at the University Hospital Mohammed VI, in Oujda, from October 2018 to March 2020. Out of 45 patients with secondary otitis, 30 had gone ossiculoplasty using endoscopic transmeatal approach. These patients were enrolled in the study. The average age of patients was 31.8 years, with a F/M sex ratio of 1.5. The examination of the eardrum showed type A otitis (18 cases) type B otitis (5 cases), type C otitis (3 cases) and type D otitis (4 cases), according to Austin classification; 70% of patients who had undergone surgery had hearing loss between 30 and 40 dB and 30% of patients had hearing loss of ≥40 dB. Type II ossiculoplasty was performed in 26 cases, using tragal cartilage in 18 cases. Autologous incus interposition graft and placement of titanium PORP were performed in 4 cases each, whereas ossiculoplasty type III was performed in 4 cases, with placement of titanium TORP. Type II ossiculoplasty group had a hearing gain of ≥20 dB (all cases), whereas type III ossiculoplasty group had a hearing gain of ≥1 dB (1 case). Success rate was 90%. The analysis of these results shows that the overall outcome is slightly worse in patients undergoing type III ossiculoplasty than in those undergoing type II ossiculoplasty. However, comparative studies between the placement of prostheses and autografts or between different types of materials have only shown minor differences reported by the same author or the same team. Many authors highlighted that the preservation of the handle of malleus could provide better functional results.

## Introduction

L´otite séquellaire constitue le stade terminal le plus favorable des otites moyennes chroniques et la forme clinique sans doute la plus fréquemment rencontrée. Les séquelles d´otite sont en principe stables et de deux faits: la perforation tympanique qui favorise les accidents infectieux aigus et les lésions tympaniques et ossiculaires qui peuvent se combiner à d´autres formes cliniques qui vont évoluer pour leur propre compte. Elles relèvent de la chirurgie dite fonctionnelle.

Pour le tympan, une myringoplastie peut être proposée avec de grandes chances de succès. Pour les osselets, l´ossiculoplastie rétablit l´effet columellaire par transposition d´osselets ou d´autres matériaux avec des résultats spectaculaires. Le but de notre travail est d´évaluer les résultats fonctionnels des ossiculoplasties en fonction du type d´ossiculoplastie et du matériel utilisé et de les comparer à ceux d´autres séries.

## Méthodes

Nous avons mené une étude rétrospective, au sein du service d´ORL et CCF au CHU Mohammed VI d´Oujda, étalée sur une durée de un an et demie, depuis le mois d´octobre 2018 au mois de mars 2020, ayant pu reconçevoir 30 patients parmi 45 otites séquellaires qui ont présenté une lyse ossiculaire et qui ont été opérés pour ossiculoplastie par une voie transméatale endoscopique. Pour la classification de la lyse ossiculaire, on a adopté la classification d´Austin en 4 types: type A: M+, S+: lyse d´une partie ou de la totalité de l´enclume; type B: M+, S-: lyse de l´enclume et de la superstructure de l´étrier, mais avec présence du manche du marteau; type C: M-, S+: lyse de l´enclume et du marteau, mais avec présence de la superstructure de l´étrier; type D: M-, S-: lyse du marteau, de l´enclume et de la superstructure de l´étrier, mais la platine de l´étrier est mobile.

Pour le type d´ossiculoplastie, on a adopté la classification de Portman: tympanoplastie type I; il s´agit de toutes les interventions ayant pour but de restaurer la seule membrane tympanique. Les osselets, les fenêtres et la trompe d´Eustache doivent être saints; tympanoplastie avec ossiculoplastie type II: il s´agit d´un malade présentant un étrier normal sur lequel on ajoute un fragment ossiculaire nouveau, soit par transposition d´un élément d´osselet, soit par la mise en place d´un fragment de cartilage, d´osselet ou d´os, soit par opération de reconstruction partielle des osselets (PORP); tympanoplastie avec ossiculoplastie type III: seule la platine est normale, un fragment ossiculaire ou TORP assure le contact entre celle-ci et le tympan. Le recueil et l´analyse des données sont faits par le logiciel SPSS 21,0.

## Résultats

**Figure 1:**
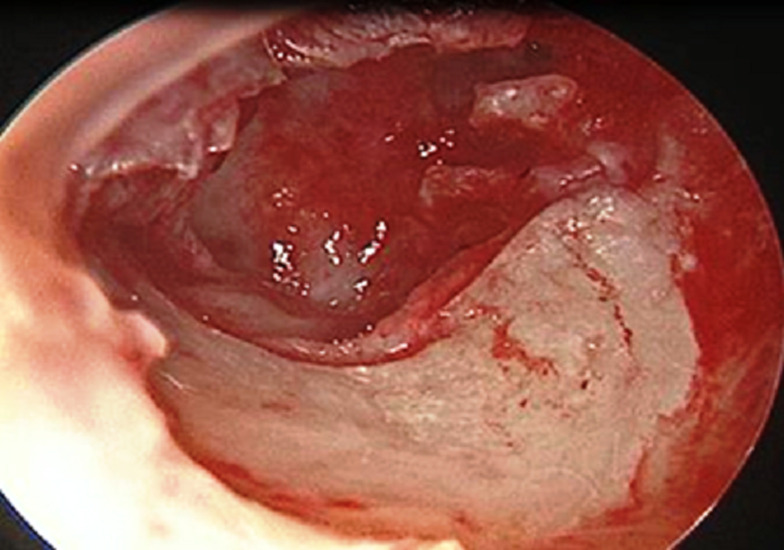
perforation subtotale gauche avec lyse du manche du marteau

**Figure 2:**
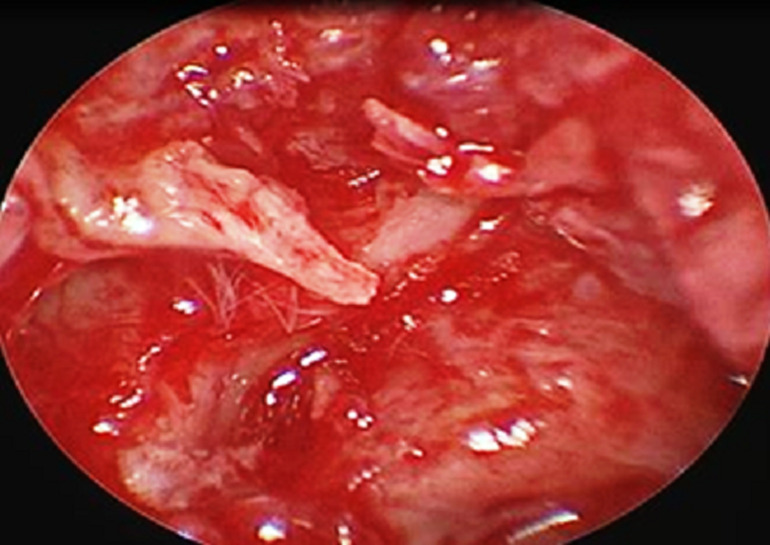
perforation subtotale gauche avec lyse de la branche descendante de l'enclume et de l'étrier

La moyenne d´âge était de 31,8 ans ± 14,77, avec des extrêmes d´âge allant de 8 ans à 57 ans avec une légère prédominance féminine: 18 femmes et 12 hommes. L´otite à répétition était la plus présente dans les antécédents dans 20 cas. L´hypoacousie était le motif de consultation le plus fréquent dans 22 cas. La perforation tympanique était gauche dans 21 cas, droite dans 9 cas, subtotale dans 24 cas et inférieure dans 6 cas. L´exploration de la caisse du tympan a montré 18 cas type A (60%), 5 cas type B (16,66%), 3 cas type C (10%) et 4 cas type D (13,33%). L´association lyse ossiculaire et foyers de tympanosclérose a été trouvée dans 9 cas (30%). La caisse du tympan était sèche dans tous les cas (Figure 1, Figure 2).

L´audiométrie tonale était réalisée dans tous les cas. Cet examen a montré une surdité de transmission unilatérale dans 27 cas, bilatérale dans 3 cas. Dans notre série, 70% des oreilles opérées avaient un Rinne audiométrique pré-opératoire entre 30 et 40 dB et 30% des patients avaient un Rinne audiométrique pré-opératoires ≥40 dB. La TDM des rochers n´a pas été demandée systématiquement sauf si l´examen otoscopique ne permet pas de préciser le type et le siège de l´atteinte ossiculaire en cas d´hypoacousie dont le facteur transmissionnel dépasse 30 dB. Elle permet de préciser l´existence de lyse ou d´ankylose ossiculaire et la présence de plaques de tympanosclérose.

**Figure 3:**
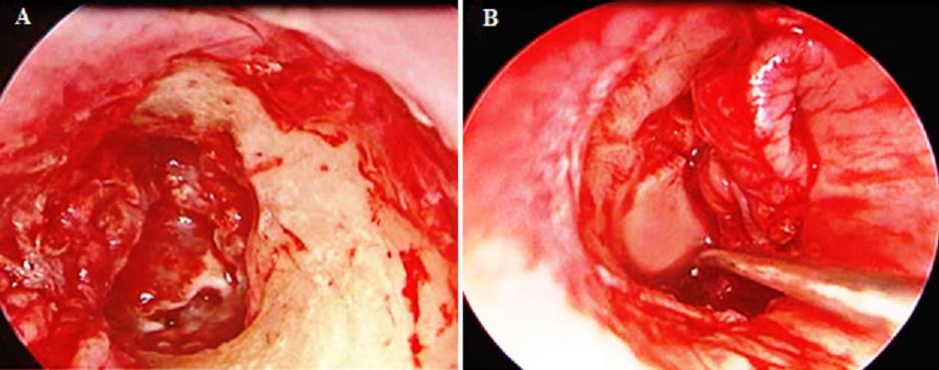
images peropératoires: (A,B) montrant une ossiculoplastie type II par interposition de greffe cartilagineuse

**Figure 4:**
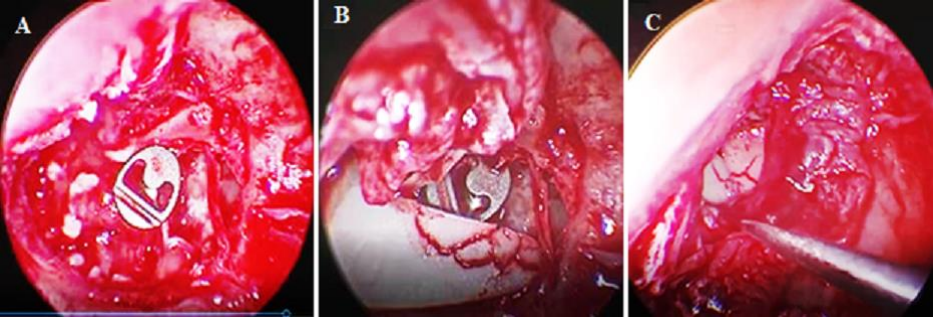
images peropératoires: (A,B,C) montrant la pose d'une prothèse ossiculaire en titane type PORP dans une ossiculoplastie type II

L´intervention s´est déroulée sous anesthésie générale dans tous les cas avec une hypotension contrôlée et infiltration locale du conduit auditif externe à la xylocaïne adrénalinée et a été menée par voie transméatale endoscopique. Dans notre série, l´intervention était unilatérale. La tympanoplastie type I ou myringoplastie a été pratiquée chez tous les patients. Il s´agit d´un geste de fermeture et de reconstruction de la membrane tympanique dont l´intérêt est double: fermer la caisse pour éviter l´infection et améliorer l´audition. L´ossiculoplastie type II a été la plus pratiquée dans 26 cas avec utilisation du cartilage tragal dans 18 cas, interposition d´enclume autologue et mise en place d´une PORP en titane dans 4 cas pour chacune alors que l´ossiculoplastie type III a été pratiquée dans 4 cas avec mise en place d´une TORP en titane (Figure 3, Figure 4).

La surveillance des malades opérés débute en post-opératoire immédiat et se poursuit après leur sortie de l´hôpital par la demande systématique d´examen acoumétrique et audiométrique afin d´évaluer les résultats fonctionnels. Dans notre série, le recul s´étend de 8 mois à 24 mois, le recul moyen étant de 12 mois. Le Rinne audiométrique post-opératoire moyen était de 12,13 dB avec des extrêmes allant de 0 à 40 dB: entre 0 et 10 dB dans 17 cas (56,66%) et entre 10 et 20 dB dans 9 cas (30%). Les gains auditifs sont alors évalués par le calcul du gain auditif moyen obtenu en se référant aux résultats du Rinne audiométrique pré et post-opératoire. Le groupe d´ossiculoplastie type II avait un gain auditif ≥20 dB dans tous les cas alors que celui d´ossiculoplastie type III ne l´avait que dans un seul cas (Tableau 1).

**Tableau 1:** gain auditif moyen en dB

Gain audiométrique moyen (dB)	Nombre de cas	Fréquence %
≤20	3	10
≥20	27	90

Au sein du groupe d´ossiculoplastie type II, on a constaté que le gain auditif dans le sous-groupe utilisant l´autologue d´enclume était supérieur à celui utilisant le cartilage tragal et la mise en place de PORP: 3 parmi 4 cas dans le sous-groupe utilisant l´autologue d´enclume avaient un gain auditif ≥30 dB alors que 3 et 2 cas dans les sous-groupes utilisant successivement le cartilage tragal et la PORP avaient un gain auditif ≥30 dB (Tableau 2).

**Tableau 2:** gain auditif moyen en dB dans le groupe d'ossiculoplastie type II selon le matériel utilisé

	Cartilage tragal	Enclume autologue	PORP
Gain audiométrique moyen (dB)	Nombre de cas	Nombre de cas	Nombre de cas
21-30	15	1	2
≥30	3	3	2

## Discussion

Les premières tympanoplasties ont été décrites par Wullstein H en 1952 [1] et Zollner F en 1954 [2], qui en distinguaient cinq types: lésion tympanique à chaîne intacte, lésion de chaîne à étrier intact, lésion de chaîne à étrier absent, protection de la fenêtre ronde sur platine mobile et fenestration du canal latéral. Actuellement, seuls les trois premiers types sont utilisés. La reconstruction de la chaîne ossiculaire ou ossiculoplastie [3] serait souhaitable dans 40 à 90% des tympanoplasties.

Les habitudes chirurgicales en matière de l´ossiculoplastie ont été bouleversées ces dernières années. Bien qu´aucune épreuve de contamination humaine par un virus ou un prion n'ait pu être observée à la suite d'une intervention de chirurgie de l'oreille moyenne, l'application du principe de précaution a fait abandonner les allogreffes et xénogreffes par la majorité des otologistes. La biocompatibilité des matériaux récents que sont l'hydroxyapatite et le titane permet d'espérer une tolérance à long terme voisine de celle des osselets, d'autogreffes ou d'allogreffes. Pour les principaux matériaux utilisés en ossiculoplastie, on distingue: les autogreffes (osselet, os cortical, cartilage), les allo et xénogreffes et en fin les prothèses ossiculaires.

L´autogreffe d´osselet est le matériau le plus utilisé en otologie vu sa biocompatibilité parfaite. Les osselets les plus utilisés sont les restes d'enclume et la tête du marteau. Le seul inconvénient de l´osselet d´autogreffe, spécifique au cholestéatome, est constitué par la possibilité d'envahissement en profondeur par la matrice épithéliale [4]. L´autogreffe d´enclume a été utilisée dans 4 cas de notre série, soit 13,33%. La corticale mastoïdienne semble être le meilleur site donneur concernant l´os cortical, vue la grande facilité de prélèvement sur le site opératoire, la bonne intégration avec couverture muqueuse et la néoformation osseuse [5].

Le cartilage tragal ou conchal, a été largement utilisé pour la réparation du cadre osseux et la myringoplastie. Les avantages sont la facilité de modelage, la disponibilité, le coût, la biocompatibilité, et le faible taux d'extrusion. Toutefois, le temps opératoire peut être prolongé, car le cartilage doit être prélevé et moulé. Le cartilage tragal a été le plus utilisé dans 18 cas de notre série, soit 60%.

Au sein des prothèses ossiculaires, on distingue plusieurs types: les plastiques poreux (proplast, plastipore, polycèle) qui se sont révélés mal tolérés avec des taux d'extrusion atteignant 30% même avec une interposition cartilagineuse [6], les céramiques bioréactives (Céravital©, Bioglass©, Macor©) ou bio-inertes (Frialite©) qui ont apporté une meilleure tolérance et des résultats fonctionnels supérieurs. Cependant, les résultats fonctionnels se sont détériorés avec le temps, faisant apparaître des lyses et des extrusions [7,8]. Seule l'hydroxyapatite apparaît donner des résultats fonctionnels proches de ceux des osselets sans détérioration avec le temps et avec des taux d'extrusion variant entre 0 et 4% selon les montages [9] et les prothèses métalliques bio-inertes n'entraînent pas de réaction tissulaire de la part de l'hôte. Ces propriétés ont été et sont toujours utilisées pour la réalisation de prothèses composites. Le titane est aujourd'hui largement utilisé pour des prothèses PORP et TORP en raison de son excellente biocompatibilité [10]. Toutefois, le titane, comme l´hydroxyapatite ne doit pas être placé directement sous la membrane tympanique ou la greffe conjonctive sans interposition de cartilage. Le titane permet également la fabrication de prothèses à mémoire de forme (mémométal). La PORP et la TORP ont été utilisées dans 4 cas pour chacune de notre série. Pour un recul moyen de 12 mois, on n´a noté aucun cas de lyse ou d´extrusion des prothèses ossiculaires. Toutefois, ce recul est insuffisant pour juger de leur tolérance à long terme. Cependant, le choix d´une prothèse ossiculaire est régit par certains critères qui sont la biocompatibilité, les données de la biomécanique ossiculaire, les formes, l´adaptabilité des prothèses, la masse, la rigidité, la longueur, la facilité d'utilisation, le prix de revient et la fiabilité des prothèses.

L´expérience de l´opérateur est un élément déterminant dans le choix et dans l´adaptation d´une prothèse ossiculaire pour obtenir un bon résultat durable. Nous pensons néanmoins qu'après la biocompatibilité, le respect d'un bon axe de transmission, le faible poids, le bon compromis entre souplesse et rigidité, et la qualité des ancrages sont les clés de la réussite [11]. La technique chirurgicale sera fonction des lésions rencontrées, de l'anatomie propre à chaque oreille, des habitudes de l'otologiste et des matériaux dont il dispose. Les détails techniques sont propres à chaque cas de figure et peu développés dans la littérature [12]. L´intervention est généralement faite sous anesthésie générale lors d'une tympanoplastie. Réalisée sous anesthésie locale lors d'un temps purement fonctionnel, elle permet l'appréciation directe en fin d'intervention du résultat.

L'utilisation d'un mesureur est indispensable surtout lorsqu'on dispose de prothèses à longueur fixe de façon à ne pas sortir inutilement une prothèse trop courte ou trop longue. L'apport sur place de la prothèse se fait à l'aide d'une micropince ou de l´aspirateur avec une pédale à rupture instantanée d'aspiration de type Portman. Lorsque la prothèse est dans la caisse, il convient de l'orienter et de la mettre en place à l'aide d'une pointe courbe ou de micro-crochets. Il est parfois nécessaire de soulever le manche du marteau pour glisser la prothèse en bonne position. La section du tendon du muscle du marteau est souvent utile au cours de ce geste mais ce geste est discuté, favorisant la latéralisation tympanique. Il convient de vérifier la bonne stabilité du montage. Une bonne précaution consiste après avoir rabattu le lambeau tympano-méatal à soulever de nouveau celui-ci pour vérifier que ce geste n´a pas provoqué la luxation de la prothèse. Enfin, il faut vérifier que la prothèse n'a pas de contact avec les parois osseuses de la caisse pour éviter tout risque de blocage secondaire.

L´utilisation d´une colle biologique (Tissucol©, Besiplast©) permet parfois de stabiliser le montage, surtout si l'on utilise une lamelle de cartilage. Il ne faut toutefois pas trop compter sur la colle pour obtenir un montage stable car l'action de celle-ci se termine après la phase de fibrinolyse (une quinzaine de jours). Il est très difficile d'analyser des séries homogènes et comparables permettant une évaluation des techniques d´ossiculoplastie et des qualités du matériel employé. S´il on y ajoute la variabilité de l'expression des résultats (gain auditif, Rinne résiduel), il devient vite difficile de comparer entre elles les différents types [13]. Certaines causes d'échecs ne sont pas dues à l'ossiculoplastie. Ce sont notamment l´évolution de la maladie otitique, la survenue d'une perforation tympanique, la latéralisation de la greffe, le comblement de l'angle tympano-méatal antérieur, les problèmes muqueux (otites fibro-adhésives) et les dysfonctionnements tubaires (épanchement).

D'autres causes d'échecs sont propres à l'ossiculoplastie elle-même, ce sont l'extrusion de la prothèse ossiculaire, le déplacement du montage avec bascule de l'osselet ou de la prothèse, la lyse de l'osselet transposé, le blocage du marteau, l'ankylose de l'étrier méconnue ou secondaire et le contact de la pièce ossiculaire avec les parois. Dans l'otite chronique simple, le Rinne résiduel est compris entre 0 et 20 dB dans 73% des cas quand l'étrier et le marteau sont présents, 60% des cas en l'absence d'étrier, 57% des cas en l'absence de marteau [14].

Nous présentons dans le Tableau 3 les résultats de 8 grandes statistiques rapportées par Merchant [15]. L'analyse de ces résultats montre que globalement les résultats sont légèrement moins bons dans les ossiculoplasties type III (TORP) que dans les ossiculoplasties type II (PORP) et que par ailleurs ils n'ont guère progressé en 30 ans malgré les recherches et l'amélioration des matériaux proposés. Cela concorde avec les résultats de notre série: on avait un gain auditif ≥20 dB dans le groupe d´ossiculoplastie type II dans tous les cas, alors qu´on avait un gain auditif ≥20 dB que dans un seul cas dans le groupe d´ossiculoplastie type III. Le taux de réussite était de 90%, conformément aux données de la littérature. De même, on avait un Rinne audiométrique post-opératoire ≥20 dB dans 86,66% des cas, un taux légèrement supérieur à celui de la littérature puisqu´il varie entre 24% pour Lau et Tos et 40-80% pour Colletti *et al*. cela pourrait être expliqué par l´étroitesse de l´effectif de notre série (Tableau 3).

**Tableau 3:** résultats auditifs après ossiculoplastie

Auteurs	NB de cas	Petites columelles y compris PORP	Grandes columelles y compris TORP
Lee et Schuknecht, 1971	936	40%	
Pennington 1973	246	70%	
Jackson, Glasscock *et al*.,1983	417	64%	43%
Brackmann, Sheehy et Luxford, 1984	1042	73%	55%
Lau et Tos, 1986	229	24%	40%
Ragheb, Gantz et McCabe, 1987	455	52%	37%
Colletti *et al*. 1987	832	48%-80%	28%-70%
Goldenberg, 1992	262	57%	58%

% de cas avec un Rinne post-opératoire ≤20dB; les résultats varient selon le recul post-opératoire: ils s'aggravent avec le temps

Les études comparatives entre différents montages [16], entre prothèses et autogreffes ou entre différents types de matériaux [6,17] n'objectivent que des différences minimes pour un même auteur ou pour une même équipe. Dans notre série, on a obtenu les meilleurs résultats avec l´interposition d´enclume autologue. Au sein du groupe d´ossiculoplastie type II, on a constaté que le gain auditif dans le sous-groupe utilisant l´autologue d´enclume était supérieur à celui utilisant le cartilage tragal et la PORP: 3 parmi 4 cas dans le sous-groupe utilisant l´autologue d´enclume avaient un gain auditif ≥30 dB alors que 3 et 2 cas dans les sous-groupes utilisant successivement le cartilage tragal et la PORP avaient un gain auditif ≥30 dB. De même, les résultats obtenus chez les cas types A et B étaient meilleurs que ceux obtenus chez les cas type C et D. Cela rejoint l´avis de beaucoup d'auteurs qui pour eux il semble que la préservation du manche du marteau permette de meilleurs résultats fonctionnels [18], mais certains optent pour un sacrifice délibéré de cet osselet dans le cholestéatome [19].

## Conclusion

L'ossiculoplastie est une chirurgie difficile qui ne manque pas de nous rappeler souvent à la modestie. Dans tous les cas, l'avenir de l'oreille à long terme doit prévaloir sur le résultat immédiat qui ne fait bien souvent que satisfaire l'orgueil du chirurgien. Enfin, les résultats des ossiculoplasties sont très «opérateurs dépendants»: chaque chirurgien agit selon ses habitudes, en fonction de son expérience et des différents cas de figure qu'il peut rencontrer.

### Etat des connaissances sur le sujet

- L´ossiculoplastie rétablit l´effet columellaire par transposition d´osselets ou d´autres matériaux avec des résultats spectaculaires;
- Les résultats sont légèrement moins bons dans les ossiculoplasties type III (TORP) que dans les ossiculoplasties type II (PORP);
- Les études comparatives entre différents montages, entre prothèses et autogreffes ou entre différents types de matériaux n'objectivent que des différences minimes pour un même auteur ou pour une même équipe.

### Contribution de notre étude à la connaissance

- Notre travail est, à notre connaissance, le premier traitant de l´ossiculoplastie dans la région de l´Oriental;
- L´autogreffe d´osselet est le matériau qui donne les meilleurs résultats fonctionnels;
- La préservation du manche du marteau permet de meilleurs résultats fonctionnels.
